# Assessment of fracture risk tools in care home residents: a multi-centre observational pilot study

**DOI:** 10.1007/s41999-020-00383-2

**Published:** 2020-10-27

**Authors:** F. Ihama, A. Pandyan, C. Roffe

**Affiliations:** 1 Department of Medicine/Elderly Care, Pilgrim Hospital Boston, Sibsey Road, Boston, PE21 9QS UK; 2 School of Allied Health Professions, Mackay Building, Keele University, Keele, ST5 5BG UK; 3 Guy Hilton Research Centre, 1 Thornburrow Drive, Stoke–on–Trent, ST4 7QB UK

**Keywords:** Fragility fracture, Tools, Risk assessment, Care home residents

## Abstract

**Background:**

Fragility fractures are common in care home residents but established tools have not been tested in this population. Aim:To identify the most practicable tool for use.

**Methods:**

**Design:**

Multicentre prospective observational cohort pilot study. Setting: 18 care homes in Boston, UK. Assessments: fragility risk score at baseline with FRAX, QFractureScore, Garvan nomogram, body mass index and TUGT for each participant. Outcomes: falls, fractures, combined falls & fractures. Follow-up; 12 months.

**Results:**

217/618 (35%) residents in the 18 care homes were enrolled. 147 (68%) had mental capacity,70 (32%) did not. There were 325 falls and 10 fractures in participants during the study. At the same time there were 1671 falls and 103 fractures in residents not participating in the study. Multiple regression analyses showed that only age had a statistically significant association with falls (*χ*^2^(1) = 5.7775, *p* = 0.0162), fractures (*χ*^2^(1) = 4.7269, *p* = 0.0297) and combined falls & fractures (*χ*^2^(1) = 4.7269, *p* = 0.0297). C-statistics were: falls; FRAX 0.544, BMI 0.610, QFractureScore 0.554, Garvan nomogram 0.579, TUGT 0.656, fractures; FRAX 0.655, BMI 0.708, QFractureScore 0.736, Garvan nomogram 0.712, TUGT 0.590, combined falls and fractures, c-statistics were same as for fractures. Fifty-four participants (25%) died during follow-up. Charlson comorbidity index predicted mortality, *R*^2^ = 0.021 (*p* = 0.034).

**Conclusions:**

QFractureScore, BMI and Garvan nomogram were good predictors of fractures and combined falls and fractures Only age had statistically significant association with the outcomes. No tool was good predictor of falls.

## Key summary points

**Aim:**

To compare the efficacy of four fragility risk assessment tools and the Timed Up and Go test (TUGT) in care home residents.

**Findings:**

None of the tools was reliable for predicting falls. The QFractureScore, BMI and the Garvan nomogram were the best predictors of fractures and combined falls & fractures. In the multiple logistic regression analyses, age was the only statistically significant covariate associated with falls, fractures and combined falls & fractures.

**Message:**

Three of the five tools tested predicted fragility fractures in the care home residents. Of these, the BMI is easiest to use, and is therefore most suitable for this population.

## Introduction

Fragility fractures are common in older people. Globally there were an estimated 9 million new fractures in the year 2000 [1]. One in two women and one in five men over the age of 50 years are likely to sustain a fracture in their lifetime [2]. With the anticipated exponential increase of the ageing population, more older persons will be admitted to care homes and fragility fractures are expected to increase in this cohort. Consequently, a well-coordinated, systematic global approach at primary prevention through risk assessment is needed because once a fragility fracture has occurred secondary prevention is less effective [3]. Guidance for prevention is given in the Blue Book published by the British Geriatrics Society [4], by National Institute for Health and Care Excellence [5] and by the NHS Right Care programme [6]. These include recommendations for service organization, pharmacological treatments, and therapy interventions. Several screening tools are available, but FRAX [7], QFractureScore [8] and Garvan nomogram [9] were the most commonly used and body mass index (BMI) proposed as a futher more simple predictor [10]. None of the fragility fracture risk assessment tool had previously been examined in care home residents. The aim of this pilot project was to identify which of these tools is most suitable for use in care home residents.

## Methods

This was a multicenter prospective observational cohort study of the ability of four standard fragility tools and the TUGT to predict falls and fractures in care homes. Care homes included residential homes (social and personal care), nursing homes (social, personal, and 24 hour nursing care), and adult disability homes (residential homes for adults with learning disabilities). Ethical approval was obtained from the Nottingham Research Ethics Committee on the 09 January 2015 (reference:14/EM/1225).

### Setting

Participants were recruited from all 18 care homes (13 residential homes, 4 nursing homes, and 1 home for adults with disability) in Boston, Lincolnshire, UK. All homes, except for the adult disability home accepted residents with dementia. Boston is a semi-rural town situated on the East coast of England in the county of Lincolnshire. The local population is older than the national average with 21% aged above 64 years compared with 18% nationally [11]. In the 2011 census, the Borough of Boston had a population of 64,000 with 15% of the population born outside the UK and 11% in the European Union accession countries such as Poland and Lithuania. The non-white population made up 2.4% of the total population in 2011 [11].

### Participants

All residents in the participating care homes were eligible for the study unless they were on the end of life care pathway. Informed written consent was obtained from the resident if they were mentally competent or from a consultee (a person who is empowered with Lasting Power of Attorney) if otherwise. Residents who were unable to consent themselves and where consent could not be obtained from a consultee were excluded from the study.

### Outcome measures

Outcome measures included falls, fractures, combined falls and fractures. *A fall* was defined as an unexpected event in which the participant came to rest on the floor or lower lower surface [12]. It was not necessary for the fall to be observed to be counted as an incident. *Fractures* were defined as break in the continuity of the bone, verified by x-ray and reported so by a Radiologist. *A fragility fracture* was defined as one sustained after low trauma [13] quantified by the World Health Organisation (WHO) as forces equivalent to a fall from a standing height or less. Vertebral fractures may occur without a fall. Skull fractures, facial fractures, fractures resulting from road traffic accidents and pathological fractures were excluded.

### Baseline assessments

The baseline assessment of fracture risk was via a structured composite questionnaire that captured all the covariates in each of the risk assessment tools as they appeared in the pdf versions; (FRAX[without bone mineral density estimate], QFractureScore-2016, Garvan nomogram, and BMI), the Timed Up & Go Test (TUGT) falls risk assessment tool [14], and the Charlson Comorbidity Index (CCI) [15]. The TUGT is a standard falls risk assessment tool which was included for comparision. It accesses gait and balance and it is recommended for falls assessment in primary care settings [16, 17]. The CCI was included to guide treatment decisions given the relatively short life expectancy of care home residents [18].

For FRAX, QFractureScore and Garvan nomogram, a 10-year fracture probability estimate of 20% or above for major fracture is recommended for therapeutic intervention [19]. The World Health Organisation (WHO) criteria for normal range of BMI of between 18.5 and 24.9 kg/m^2^ [20] was used for the following reasons; a care facility that catered for younger adults with learning disability was included in the study, some of the care homes catered for relatively young adults with comorbidities such as dementia. For the TUGT, people who take longer than 12 s to complete are at high risk of falls [21]. There are no publications for the cut-off values for the CCI.

Body weight of each participant who was ambulant was measured by standard Seca weighing scale with the participant wearing light clothes with both feet off the ground. For the participants who were bed bound, their weight were estimated using standard digital hoist. Height estimation for the participants who were ambulant were done by wall-mounted scales and for those who were chair-bound, bed-bound or severely infirm, the height were estimated using the ulna length [22]. These measurements were taken at the left forearm with a tape measure from the point of the elbow (olecranon process) and the midpoint of the wrist (styloid process). If the left forearm was not accessible or was deformed by previous fracture or disease, the right forearm was used instead. BMI was calculated by dividing the weight in kilograms by the height in meters squared. For the TUGT each participant was given one practice trial and then three tests were taken and the mean duration to complete the test was calculated. Participants were allowed to use any walking aids and/or be could assisted by a carer if needed. The CCI was calculated online (farmacologiaclincs.info) for each participant.

### Follow-up

The follow-up was for 12 months after enrolment. Every month within that period, anonymized data of the number of falls, fractures and death were obtained for each participant from the incident book in each care facility. The incidents were recorded as the total number at the end of follow-up. The duration of 12 month follow-up was chosen because of the high mortality of care home residents. Deaths were verified from the general practitioners` (GP) register and included in the analyses. If a participant was lost to follow-up, it was assumed that they were alive and did not have any falls or fractures.

### Data analysis

Data were imported and analyzed using SAS version 9.4 (SAS Inc., Cary, NC). Descriptive statistics and frequency tables were used to summarize the study variables. Three multiple logistic regressions [23] were performed to investigate if there was a relationship between each of the outcome variables, falls, fractures, and combined falls and fractures, and the 27 predictors of interest. Due to the issue of separation, Firth’s penalized likelihood approach [24, 25] was implemented to reduce bias in the parameter estimates. Separation means that the responses can be perfectly separated by a single factor or by a combination of factors.

Wald chi-square tests for type III analyses were used to determine if the effects of predictors were statistically significant. The Hosmer–Lemeshow goodness-of-fit test [23] was used to determine the model adequacy (*p* value > 0.05 indicates good model fit). The odds ratio estimates and the corresponding 95% confidence intervals (CI) were used to quantify the strength of the effects for the predictors.

Fifteen logistic regressions with one predictor (one of the tools: FRAX, QFractureScores, Garvan nomogram, BMI, and TUGT) and one control variable (mortality) were performed and the predictive power of each model was evaluated using the concordance index (c-index or c-statistic) [26]. The c-index estimates the probability that the predictions and the outcomes are concordant. C-index is equal to the area under the receiver operating characteristic (ROC) curve and ranges from 0.5 to 1, with a value of 0.5 indicating predictions were no better than random guessing, 0.7 indicate a good model, 0.8 and over indicate a strong model and a value of 1 indicate a perfect model [27]. For any tests, a p value less than 0.05 indicated statistical significance.

## Results

There were 618 residents in the 18 care homes in total out of which 217 (35%) were enrolled in the study and 401 (65%) could not be recruited. The most common reason for non-enrolment was inability to gain consent from a consultee in residents not competent to consent themselves (*n* = 263, 66%). Just under a third (*n* = 111, 28%) were competent but decided not to participate, and few (*n* = 24, 6%) were on end-of-life pathway.

The number of residents with mental capacity in all the 18 care homes was 258/618 (42%), and 57% (147/258) provided informed consent. There were 333 residents without mental capacity (54% of 618), and in 21% (70/333) consultee consent could be obtained. Thus only 217 of 618 care home residents (35%) could be enrolled in the study. Of these 147 (68%) had mental capacity and 70 (32%) did not.

Table 1 summarizes the baseline characteristics of the participants and the five tools (10 year absolute probability by FRAX, 10 year fracture probability by QfractureScore, 10 year absolute fracture probability by Garvan nomogram, BMI and TUGT). The majority (61.8%) of the participants were females and all the participants (100%) were Caucasian. The majority (97.7%) did not take alcohol in excess and only few (4.2%) smoked cigarette. Most (81.2%) participants were in residential settings. There were 1671 falls in the residents who were not included in the study (3.4 falls/resident/year) and 325 falls in the participants (1.5 falls/participant/year). There were 103 fractures in the residents who were not included in the study (0.2 fractures/resident /year) and 10 fractures in the participants (0.05 fractures/participant/year). Among the 217 participants, 43% had falls, 4.6% had fractures, and 4.6% had both falls and fractures. Approximately a quarter (24%) died during the study. The mean CCI was 30.6% (SD 20.7) for all the participants and 36% (SD 21.1) in those who died.

**Table 1:** Descriptive statistics of baseline characteristics, three continuous predictors and five tools (*N* = 217)

Characteristics	No. participants, (%)
*Gender*	
Male Female	83 (38.2) 134 (61.8)
*Ethnicity*	
Caucasian Others	217 (100) 0 (0)
*Alcohol*	
≥ 3 units a day < 3 units a day	5 (2.3) 212 (97.7)
*Smoking*	
Smoker Non-Smoker	9 (4.2) 208 (95.8)
*Abode*	
Residential Nursing Home	177 (81.6) 40 (18.4)

	Mean (SD)	Min	Max
*Predictors*			
Age in years	81.21 (12.51)	36.00	107.00
Number of comorbidities	3.30 (1.76)	0	10.00
Charlson`s comorbidity index for 1 year	30.65 (20.75)	12.00	85.00
*Tools*			
10 year absolute probability by FRAX	19.47 (11.99)	1.90	67.00
Body mass index (BMI)	24.26 (7.21)	13.40	58.00
10 year fracture probability by QfractureScore	35.77 (26.48)	1.10	99.90
10 year absolute fracture probability by Garvan nomogram	42.16 (27.84)	0.60	100.00
Timed Up and Go Test (TUGT)	33.80 (23.37)	9.00	126.00

*N *124 for TUGT

Interpretation: Table 1 shows that the majority of the participants were females, and all were Caucasian. The majority did not take alcohol in excess or smoke cigarette. The participants were recruited mostly from residential homes and they were elderly and frail with multimorbidities. The CCI predicted that about a third would not be alive in the following 12 months. The 10-year absolute fracture probability differed. Both QfractureScores and Garvan nomogram score indicated treatment for osteoporosis but not by the score with FRAX. The mean BMI of the participants suggested that they were well nourished. The majority had difficulty with mobility

Table 2 shows the results of the three multiple logistic regressions used to investigate if there was a relationship between each of the outcome variables, falls, fractures, and combined falls and fractures, and the predictors of interest. Only 26 predictors were used in the analyses as one predictor, rheumatoid arthritis, had only 1 type of response (data of rheumatoid arthritis for all subjects were “No”).

**Table 2:** Predictors of Falls and Fractures

Variable	Falls				Fractures			
	OR	Lower 95% CI	Upper 95% CI	*p *value	OR	Lower 95% CI	Upper 95% CI	*p *value
Intercept				0.6149				0.6395
Sex	0.671	0.353	1.278	0.2254	0.919	0.262	3.227	0.8949
Previous fracture	1.022	0.551	1.895	0.9453	0.688	0.224	2.110	0.5133
Parental fractured hip	0.553	0.110	2.781	0.4722	0.506	0.020	12.951	0.6804
Current smoking	0.630	0.195	2.033	0.4397	0.255	0.046	1.423	0.1194
Glucocorticoids	2.018	0.190	21.390	0.5598	3.583	0.027	482.119	0.6099
Secondary osteoporosis	0.448	0.022	9.129	0.6013	0.143	0.002	10.736	0.3777
Alcohol 3 or more units/day	8.073	0.281	232.259	0.2230	2.586	0.025	264.675	0.6874
Diabetes	1.556	0.674	3.591	0.3004	1.470	0.258	8.362	0.6639
Osteoporosis/hip fracture in a parent	0.990	0.185	5.290	0.9910	1.332	0.044	40.253	0.8690
live in a nursing or care home	1.106	0.060	20.421	0.9460	6.875	0.099	475.044	0.3724
History of falls	0.551	0.264	1.153	0.1135	0.319	0.058	1.743	0.1872
Dementia	1.425	0.738	2.750	0.2911	0.803	0.242	2.663	0.7201
Cancer	0.752	0.253	2.230	0.6068	1.517	0.178	12.965	0.7033
Asthma or COPD	1.239	0.355	4.317	0.7370	0.187	0.029	1.191	0.0759
Heart attack, angina, stroke or TIA?	1.185	0.558	2.519	0.6584	0.413	0.104	1.650	0.2111
Chronic liver disease	0.386	0.039	3.847	0.4171	0.084	0.005	1.541	0.0951
Chronic kidney disease	1.022	0.328	3.183	0.9697	1.774	0.154	20.397	0.6454
Parkinson`s disease	0.300	0.061	1.468	0.1372	0.272	0.031	2.364	0.2380
Malabsorption (ex: Crohns disease)	0.596	0.059	6.029	0.6610	0.081	0.002	2.910	0.1687
Endocrine problems	1.887	0.053	66.798	0.7271	0.563	0.008	37.395	0.7884
Epilepsy or taking anticonvulsants	0.692	0.293	1.633	0.4008	0.433	0.077	2.428	0.3414
Taking antidepressants	0.987	0.509	1.913	0.9688	1.500	0.366	6.149	0.5729
Taking steroid tablets regularly	0.786	0.049	12.620	0.8648	0.290	0.003	31.636	0.6053
Age	**1.036**	**1.007**	**1.067**	**0.0162**	**1.083**	**1.008**	**1.163**	**0.0297**
Number of comorbidities	0.966	0.790	1.181	0.7331	0.735	0.480	1.125	0.1562
Charlson`s comorbidity index for 1y	1.002	0.983	1.021	0.8723	1.005	0.970	1.042	0.7685

Results of multiple logistic regression (Outcome variable = falls)

*OR *odds ratio, *CI *confidence interval, *lower *lower bound of the CI, *upper *upper bound of the CI. For sex, “male” was the reference group. For all the other categorical predictors, “yes” was the reference group. The logistic regression model was modeling the probability of the outcome variable (falls and fractures, respectively) being “Yes”. Hosmer and Lemeshow goodness-of-fit (*χ*^2^(8) = 12.1608, *p* = 0.1442). c-index = 0.694 for falls and (*χ*^2^(8) = 6.3869, *p* = 0.6040). c-index = 0.851 for fractures

Interpretation: In his study of 217 residents recruited from all 18 care homes in Boston, Lincolnshire, UK (mean age 81 yers, 62% female, mean number of comorbidities 3.3) logistic regression analysis showed that the only statistically significant predictor of falls and fractures was age (in bold) demonstrated statistically significant association with falls and fractures

From the analyses presented in Table 2, there was a statistically significant relationship between falls and age (*χ*^2^(1) = 5.7775, *p* = 0.0162; Table 3). Compared to younger subjects (i.e. age < 81.2 years), older subjects were more likely to fall (OR = 1.036, 95% CI = [1.007, 1.067]; Table 3). For a one-unit increase in age, there was about 3.6% increase in the odds of falls. There was no statistically significant relationship between falls and any of the other covariates.

**Table 3:** Results of the computation of the c-statistics of the tools

Outcome	Tool	Wald *χ*^2^	*p*	OR	95% OR		c-index	*p* for mortality
					Lower	Upper		
Falls	FRAX	0.0462	0.8299	1.002	0.980	1.025	0.544	0.6443
	BMI	5.2367	0.0221	0.953	0.914	0.993	0.610	0.8747
	QfractureScore	1.8208	0.1772	1.007	0.997	1.018	0.554	0.7805
	Garvan	3.5285	0.0603	1.010	0.999	1.020	0.579	0.8851
	TUGT	7.2964	0.0069	1.028	1.008	1.049	0.656	0.2529
Fractures	FRAX	1.1070	0.2927	1.026	0.978	1.077	0.655	0.2559
	**BMI**	**3.5488**	**0.0596**	**0.869**	**0.751**	**1.006**	**0.708**	**0.3210**
	**QfractureScore**	**3.9677**	**0.0464**	**1.023**	**1.001**	**1.046**	**0.736**	**0.3555**
	**Garvan**	**2.8181**	**0.0932**	**1.019**	**0.997**	**1.042**	**0.712**	**0.4052**
	TUGT	0.0680	0.7943	0.995	0.961	1.031	0.590	0.0638

*FRAX *10-year absolute probability by FRAX, *BMI *body mass index, *QfractureScore *10-year fracture probability by QfractureScore, *Garvan *10-year absolute fracture probability by Garvan nomogram, *TUGT* Timed Up and Go Test. *Wald χ*^2^ Wald chi-square statistic; *p*   *p* value, *OR *odds ratio, *CI *confidence interval, *lower *lower bound of the CI, upper = upper bound of the CI, *p* for mortality = *p* value for the control variable, mortality. Degrees of freedom (DF) for Wald *χ*^2^ = 1. For sex, “male” was the reference group. For all the other categorical predictors, “yes” was the reference group. The logistic regression model was modeling the probability of each outcome variable (falls, fractures, and combined falls and fractures), being “Yes”

Interpretation: For falls, none of the tools was a good predictor as their c-statistics were lower than 0.7. For fractures, BMI, QFracturescore and Garvan nomogram (in bold) were good predictors as their c-statistics were above 0.7. For combined falls and fractures, BMI, QFracturescore and Garvan nomogram (in bold) were good predictors as their c-statistics were above 0.7

The result of the analyses presented in Table 2, showed that there was a statistically significant relationship between fractures and age (*χ*^2^(1) = 4.7269, *p* = 0.0297. Compared to younger subjects, older subjects were more likely to have fractures (OR = 1.083, 95% CI = [1.008, 1.163]. For a one-unit increase in age, there was about 8.3% increase in the odds of fractures. There was no statistically significant relationship between fractures and any of the other predictors. The result of the analysis for combined falls and fractures were exactly the same as that for just fracture alone.

Table 3 shows the results of the 15 logistic regressions, each with 1 predictor (1 of the tools: FRAX, QFractureScores, Garvan nomogram, BMI, and TUGT) and 1 control variable (mortality). The effect of the control variable, mortality, was not significant (*p* > 0.05) in any of the models, indicating there was no relationship between mortality (the competing risk) and any of the outcome variables. The CCI prediction of the outcome (alive or dead) (coefficient of determination) was statistically significant; *R*^2^ = 0.021 (*p* = 0.034).

For falls, BMI (*p* = 0.0221) and TUGT (*p* = 0.0069) were statististically significant predictors. Compared to subjects with lower BMI (i.e. BMI < 24.3 kg/m^2^), subjects with higher BMI were statistically significantly less likely to fall (OR = 0.953, 95% CI = [0.914, 0.993]). Compared to subjects with lower TUGT (i.e., TUGT < 33.8 s), subjects with higher TUGT were more likely to fall (OR = 1.028, 95% CI = [1.008, 1.049]). Based on the results of the c-index, among the five tools, TUGT was the best predictor for falls (c-index = 0.656).

For fractures, 10-year fracture probability by QfractureScore (*p* = 0.0464) was a statistically significant. Compared to subjects with lower 10-year fracture probability by QfractureScore, subjects with higher 10-year fracture probability by QfractureScore (QFractureScore > 35.7%) were statistically significantly more likely to have fractures (OR = 1.023, 95% CI = [1.001, 1.046]). Based on the results of the c-index, among the five tools, the 10-year fracture probability by QfractureScore was the best predictor for fractures (c-index = 0.736).

The results for combined falls and fractures were exactly the same as that for just fracture alone. Based on the results of the c-index, among the five tools, the 10-year fracture probability by QfractureScore was the best predictor for combined falls and fractures (c-index = 0.736). But based on the c-statistics of 0.7 and above (regardless of the *p *value), three of the tools (QFracturescore, Garvan nomogram and BMI which had c-statistics of 0.7 and above) were adjudged good predictors of fractures and combined falls and fractures.

The median BMI of the participants who did not fall nor sustain incident fractures (57%, 124/217) was 24 kg/m^2^, the median BMI of the participants who fell but did not sustain incident fractures (38%, 83/217) was 21.2 kg/m^2^ and the median BMI of the participants who fell and sustained incident fractures (4.6%, 10/217) was 19.9 kg/m^2^.

**Fig. 1:**
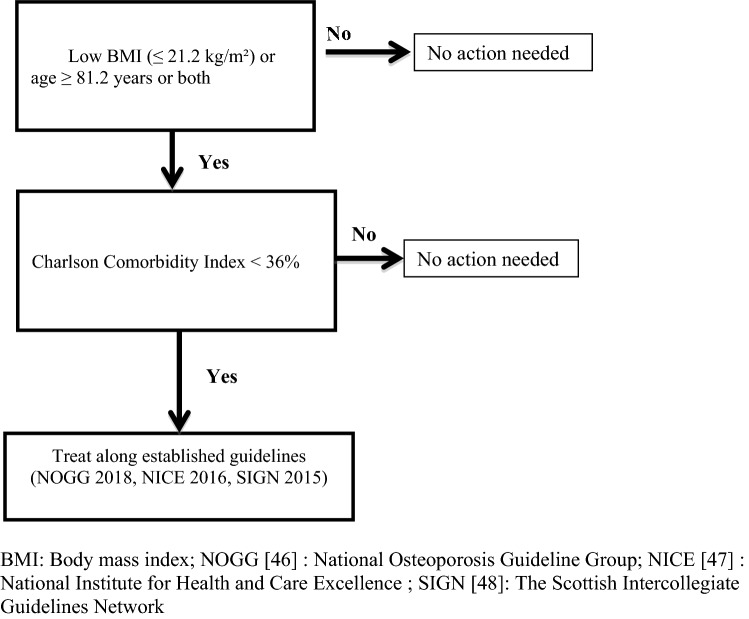
Algorithm for the management of fragility fractures in care home residents. *BMI* Body mass index, *NOGG* [46] National Osteoporosis Guideline Group, *NICE* [47] National Institute for Health and Care Excellence, *SIGN* [48] The Scottish Intercollegiate Guidelines Network. Interpretation: The algorithm utilised three continous variables (BMI, age, and CCI) and treatment guidelines in the design. It consists of three steps. The first step used BMI and age, the second step, the CCI and the third step, three treatment guidelines. The application of this algorithm is simple and within the competence of most care home staff

Using the BMI of the participants who fell but sustained no fractures (median 21.2 kg/m^2^), age of the participants (mean 81.2 years) and CCI (mean 36%) an algorithm was designed for risk identification of care home residents who were at high risk of fragility fractures (Fig. 1). Of the five tools, BMI was easiest to assess, took negligible mean time to compute (1 min; vs FRAX 1 min, Garvan nomogram 1 min, QFractureScore 2 min, TUGT 2 min) and was the only tool which could be implemented without additional training.

## Discussion

The main findings from this study were that falls were common in this cohort with an incidence of 1.5 falls per participant per year and 3.4 falls/per year for the residents not recruited but of these falls few resulted in fractures. Of the covariates, only age demonstrated statistically significant association with falls, fractures and combined falls and fractures. Using a cut-off value c-statistic of 0.7 and above to indicate good models [27], none of the tools was good at predicting falls, QFractureScore, BMI and Garvan nomgram were good predictors of fractures and combined falls and fractures.

However, not all tools were equally practicable in this population. QFractureScore has some limitations: it is web-based and it consists of many covariates making assessment cumbersome [28]. Garvan nomogram is also web-based and this is a limitation where computers are not available. The World Health Organisation Fracture Risk assessment tool, FRAX, was not considered good a predictor of falls, fractures and combined falls and fractures.

Thus BMI was chosen as the best tool to design an algorithm because it is easy to perform and within the competence of most care home staff. Also both QFractureScore and Garvan nomogram estimate fracture probability over a 10-year period but most care home residents have an average life expectancy of less than 3 years [18] which makes these estimates unrealistic. The high mortality of care home residents is supported by the findings in this study where 24% died within 12 months.

In our study, the majority (57%) of the participants had a BMI of 24.3 kg/m^2^. Thirty eight percent who had falls but no incident fractures had BMI of 21.2 kg/m^2^ and 4.6% who had falls and incident fractures had BMI of 19.9 kg/m^2^. Thus a sizeable proportion (43%) of the participants had falls and or fractures. The WHO criteria for the definition of normal BMI (18.5–24.9 kg/m^2^) are low for the elderly as they are not sensitive to the natural changes that occur in successful ageing; weight loss, sarcopenia, increase and redistribution of fat toward the abdomen, loss of bone and calcium and its consequences on height. This is supported by the ORs from the logistic regression in this study which showed that older people with BMI of 24.3 kg/m^2^ or more were less likely to fall. The recent Global Leadership in Malnutrition (GLIM) definition defines BMI of less than 20 kg/m^2^ and 22 kg/m^2^ in people under 70 years and over 70 years, respectively, as malnutrition [29]. Using the GLIM classification therefore, although the majority of the participants in our study had normal BMI, a good proportion were undernourished.

It has also been reported that elderly people who have BMI of less than 25 kg/m^2^ which is considered normal represent a group with relatively increased mortality [30]. In contrast, the lowest mortality risk from older people was observed for BMI range 25 kg/m^2^—27 kg/m^2^, which means overweight category might be appropriate for elderly people [31]. Thus it was recommended that older adults with values of less than 23 kg/m^2^ should be encouraged to gradually increase their BMI with a combination of calorie dense foods and resistance training exercise [32].

A critical quantitative analysis of three ethnic National Health and Nutrition Examination Survey (NHANES) groups in USA (non-Hispanic (NH) white, NH Black and Mexican American adults) found there was heterogeneity in body shape and composition; % fat, muscularity and trunk fat that associate with many clinical outcomes [33]. They concluded there was little to be gained by introducing race/ethnic specific BMI cut-off values using solelyadiposity as the reference. Thus in our study, the BMI values may be applicable regardless of ethnic/racial group.

Low BMI is associated with a substantial increase in fracture risk in both sexes and for all types of fragility fractures particularly hip fracture. The risk ratio is not linear [10] been higher at lower values of BMI, especially BMI of 20 kg/m^2^ or less. But at BMI of between 25 and 35 kg/m^2^, the differences in the risk ratio are small. The underlying mechanisms by which low BMI increases fracture risk are conjectural. These include greater liability to falls [34], reduced bone strength [35], nutritional deficiencies of protein, aminoacids, vitamin A, C, D, E and minerals such as iron, selenium and zinc [36]. Low BMI also results in compromised immune system which increases propensity to falls and osteoporosis [37]. There is also the effect of decreased padding over the greater trochanter which increases the risk of hip fracture following a fall.

BMI is a function of two variables, weight and height, therefore it is important to estimate both accurately. Estimation of BMI is a vital part of the care process in residents. In this study, both were measured using generally agreed methods. While the measurements of both indices does not usually pose problems in the ambulant, their estimation is more challenging for the chair-bound or bed-bound resident. For weight, hoist scales provide effective method of accurately weighing bed-bound residents [38]**.** For height, ulna length is an alternative to height in the management of osteoporosis. One study which recruited 640 patients (age range 40–90 years) to determine the effect of height measured using a stadiometer, ulna length and arm span on the assessment of the risk of fragility fracture did not show statistically significant differences in different height measurement procedures [39]. Ulna measurement is reproducible and accurate, and provides a precise predictor of height [40].

QFractureScore [8] is recommended by NICE and it includes abode as covariate which is advantagous. Fracture probability can be computed yearly for 10 years making it valuable for people with short life expectancy. QFractureScore is the most complex of the tools; it has 31 clinical risk factors in the updated version (QFracture-2016) therefore the applicability of the tool in clinical setting is questionable. The usefulness of a tool is dependent to some extent on the ease of use. Also, in the development of QFractureScore, the risk factors were only assessed at baseline, not taking into account any changes in risk factor status during follow-up. For example, a person who developed an incidental stroke would be incorrectly classified during the follow-up. The mean 10-year QfractureScore in this study was 35.8% which was above the 20% recommended for therapeutic intervention [19]. This suggests that many of the participants might benefit from treatment.

Garvan nomogram has six predictors, and when BMD is not available, body weight can be substituted. The two versions (one with BMD and the other with weight if BMD is not available) give options for application. Fracture probability can be computed for 5 years and 10 years. The mean 10-year Garvan score in this study was 42.2% which also indicates that most of the participants would benefit from treatment.

The mean 10-year fracture probability with FRAX was 19.4%, which is below the 20% recommended cut-off value for therapeutic intervention [19]. Many of the covariates in the tools did not demonstrate statistically significant association with the outcomes with the exception of age. There are three plausible explanations. The first is that this study was a pilot study and not powered. The second is that many of the covariates were actually not essential and therefore diluted the effects of BMI (which is included as a covariate) towards the null. The third is that there is difference in the sample frame. The pattern of behaviour in care home population is different from those in communities (e.g., more supervision during activities, increased time spent on sedentary activities). An attempt was made to obtain the weightings of the covariates in FRAX from the WHO headquarters in Geneva but we were informed this is a classified information. In a prospective cohort study, the Fracture Risk Epidemiology in the Frail Elderly (FREE) study which was designed to evaluate risk factors for falls and fractures in a population of 1894 older people (1433 women and 461 men) from 52 care homes and 30 hostels in Northern Sydney [41], it was found that some of the risk factors for fragility fractures in care home residents differed from those in community dwelling older people.

This is concordant with our finding that the vast majority of the covariates in the established fragility tools were not useful in this cohort of care home residents. These fragility fracture risk assessment tools were derived from relatively healthy community dwelling older people. Care home residents are a district cohort, they are frail with multiple co-morbidities and more physically, mentally and socially challenged than community dwelling older people and therefore need different risk assessment tools. Older people in care home residents are three times more likely to fall compared to older people in the community and the consequences of these incidents including fractures are also more common and serious. The mean age of the participants in this study showed that they can be categorised as ‘old old’ defined as people aged 80 years and over [42]. This represent a cohort in which falls and fractures are particularly common as shown in the multivariate analysis. This observation is concordant with previous publications [43]

Although the TUGT had the best c-statitistics for falls, it was not a good predictor of falls (c-statistics 0.656). Also, it is challenging to perform as it requires adequate space, a stop watch and the undivided attention of the examiner. In this study, 42.9% (93/217) could not undertake the test and of those who did 38% (83/217) needed the assistance of a walking aid and a carer.

The design of the algorithm in this study utilized three predictors which were considered important in the prevention of fragility fractures; BMI, age and CCI. While it is important to prevent fragility fractures in individuals with reasonable prognosis, such treatment would be futile in the residents who have shortened life expectancy. The median BMI of the participants who fell but did not sustain fractures (21.2 kg/m^2^) was used as the cut-off value because the majority of fragility fractures result from falls. The mean age of the the participants (81.2 years) was used as cut-off because that represented a sizeable proportion of the participants. The mean CCI for the participants who died during the 12 months of follow-up was 36%, thus it may be reasonable to use this as a cut-off value in the decision to offer treatment or not. Unfortunately, there are no publications to compare with. The first step of this algorithm involves both BMI and age using the values derived from the study as the cut-off. The second step in the elimination process uses the mean CCI of the participant who died during the 12-months of follow-up. The third and last step is the management of those at high risk of fragility fractures which is beyond the remit of our aim.

In this study, many residents who did not possess mental capacity and were at high risk of falling did not provide informed consent through Consultees therefore only 32% of this could be recruited. More incident falls and fractures may have been recorded if a higher proportion of residents without capacity to provide informed consent had been recruited. For example of the ten incident fractures, four (40%) occurred in this subgroup and given that the majority (54%) of the care home residents lacked mental capacity, the data are skewed. A possible solution to mitigate this limitation would be for the Government to consider including in the Lasting Power of Attorney (LPA) application the question ‘If you are admitted to a care home in the future, would you be willing to participate in research’? Research regulatory bodies such as the Health Research Authority (HRA) could modify some of the existing rules that govern participation in care home research without compromising the standards that breach research ethics. For example, the care manager could be authorised to be a proxy decision maker if the ethics committee considers the research to be non-invasive given that relatives may be more emotive and less likely to give consent for participation. Also, the waiver of consent adapted by the USA should be considered [44]. This guidance recommends public consultation. In this regard, the theme for discussion should be waiver for observational studies. A further method to facilitate research participation is to initiate the discussion with the next of kin at the point of admission to the care home. If it is felt that the resident would be willing to participate in future research, then the authority to consent could be delegated to the care home managers.

A limitation of this study is that it was impossble to obtain consent for many residents who did not have mental capacity. Most of the participants in this study had mental capacity which is not fully representative of the care home population, thus the data are skewed. It was not possible to obtain the characteristics of the residents who were not recruited for comparison of the similarities and differences because of ethical constraints. Research in care homes is challenging, none of the care facilities in this study had previously participated in studies. The majority of residents in the 18 care homes in this study lacked mental capacity and consent from consultee was obtained in only 21% and in 57% of residents who possessed mental capacity with overall consent rate of 35%. Publications on recruitment rates for mentally competent and mentally incompetent residents in care homes are sparse. Zermansky and colleagues obtained higher consent rate of 41% [45]. The difficulty in obtaining informed consent from consultees has implications for research in care home residents as the majority have dementia and cannot consent but are most likely to benefit from research findings tailored to their needs. Another limitation of this study is that participants were restricted to care home residents in one rural town with a mainly Caucasian population. The population of ethnic minority in Boston is small and this accounts for the results obtained. For example, the ethnic minority population in London was 40.2% compared with 2.4% in Boston as by the 2011 census [13]. Due to these limitations, the results of this study may not be generaliazable.

## Conclusions

In conclusion, research in care homes is important but challenging because only few residents have the mental capacity to consent. This study showed that QFracturescore, Garvan nomogram and BMI were good at predicting fractures and combined falls and fractures but in practice, only BMI was the most practicable predictive tool. BMI is easy to assess and could be used as the basis for fragility fracture risk assessment. This should be assessed formally in a larger, representative and fully powered study using the essential data from this pilot study
